# Co-reactivity pattern of glucose metabolism and blood perfusion revealing DNA mismatch repair deficiency based on PET/DCE-MRI in endometrial cancer

**DOI:** 10.1186/s40644-024-00805-5

**Published:** 2024-11-25

**Authors:** Xiaoran Li, Bixiao Cui, Shijun Wang, Min Gao, Qiuyun Xing, Huawei Liu, Jie Lu

**Affiliations:** 1 Department of Radiology and Nuclear Medicine, Xuanwu Hospital, Capital Medical University, Beijing, China; 2 Beijing Key Laboratory of Magnetic Resonance Imaging and Brain Informatics, Capital Medical University, Beijing, China; 3 Department of Obstetrics and Gynecology, Xuanwu Hospital, Capital Medical University, Beijing, China; 4 Department of Pathology, Xuanwu Hospital, Capital Medical University, Beijing, China; 5 Department of Ultrasound Diagnosis, Xuanwu Hospital, Capital Medical University, Beijing, China; 6 GE Healthcare China, Pudong New Town, Shanghai, China

**Keywords:** Endometrial cancer, [^18^F]FDG, PET, DCE-MRI, Mismatch repair deficiency

## Abstract

**Background:**

Identifying DNA mismatch repair deficiency (MMRd) is important for prognosis risk stratification in patients with early-stage endometrial cancer (EC), but there is a notable absence of cost-effective and non-invasive preoperative assessment techniques. The study explored the co-reactivity pattern of glucose metabolism and blood perfusion in EC based on hybrid [^18^F]fluorodeoxyglucose ([^18^F]FDG) PET/dynamic contrast enhanced (DCE)-MRI to provide an imaging biomarker for identifying MMRd.

**Methods:**

Patients with a history of postmenopausal bleeding and initially diagnosed with EC on ultrasound were recruited to perform a PET/DCE-MRI scan. Glucose metabolism parameters were calculated on PET, and blood perfusion parameters were calculated semi-automatically by the DCE-Tofts pharmacokinetic model. The MMRd of early-stage EC was evaluated by immunohistochemistry. The synchronous variation of PET and DCE-MRI parameters was compared between the MMRd and mismatch repair proficiency (MMRp). The association between PET/DCE-MRI and MMRd was analyzed by logistic regression to establish the digital biomarker for predicting MMRd. Receiver operating characteristic curve, decision curve analysis, and the net reclassification index (NRI) were used to evaluate the value of the digital biomarker in identifying MMRd.

**Results:**

Eighty-six early-stage EC cases (58.92 ± 10.13 years old, 34 MMRd) were enrolled. The max/mean standardized uptake value (SUV_max_/SUV_mean_), metabolic tumor volume, total lesion glycolysis, transfer constant (K_trans_), and efflux rate (K_ep_) were higher in MMRd than those in MMRp (*P* < 0.001, < 0.001, 0.002, 0.004, < 0.001, and 0.005, respectively). The correlations between glucose metabolism and blood perfusion were different between the MMRd and MMRp subgroups. SUV_max_ was correlated with K_ep_ (*r* = 0.36) in the MMRd. SUV_mean_ (odds ratio [OR] = 1.32, *P* = 0.006) and K_trans_ (OR = 1.90, *P* = 0.021) were independent risk factors for MMRd. And the digital biomarker that combined SUV_mean_ and K_trans_ outperformed in identifying MMRd in early-stage EC more than DCE-MRI (AUC: 0.83 vs. 0.78, NRI = 13%).

**Conclusion:**

A potential digital biomarker based on [^18^F]FDG PET/DCE-MRI can identify MMRd for prognosis risk stratification in early-stage EC.

**Supplementary Information:**

The online version contains supplementary material available at 10.1186/s40644-024-00805-5.

## Background

Endometrial cancer (EC) is the most common gynecological cancer in high-income areas, 65–76.7% of which presents early-stage [1–3]. DNA mismatch repair deficiency (MMRd) driven by inactivating methylation or mutation of an MMR gene (MLH1, PMS2, MSH2, or MSH6) is an important molecular subtype for EC in the clinical guideline that is associated with the risk of Lynch syndrome and prognostic risk stratification [4]. The MMRd subtype of patients with early-stage EC had lower 2-year progression-free survival (PFS = 88%) compared to that of other subtypes of EC [5]. Besides, MMRd reduced the local control of radiotherapy, but increased the response of immune checkpoint inhibitors in early-stage EC [6, 7]. Dostarlimab, a promising PD-1 inhibitor, is approved by the Food and Drug Administration (FDA) for MMRd subtype EC monotherapy, and it has been proven in the GARNET trial to have a higher objective response rate and duration of response in patients with MMRd than in patients with MMR proficiency (MMRp) [8–10]. Moreover, the phase 2 KEYNOTE-158 clinical trial has identified that the EC of the MMRd subtype had a higher objective response rate under pembrolizumab treatment, and pembrolizumab monotherapy could improve health-related quality of life in patients with MMRd EC [11–14]. Thus, pre-treatment identification of MMRd is cost-effective and can be used to guide adjuvant treatment decisions and predict prognosis in early-stage EC [15].

Currently, the main methods for testing MMRd status include immunohistochemistry (IHC) and polymerase chain reaction-based microsatellite instability. However, there was an absence of a non-invasive and cost-effective tool for revealing MMRd status in EC clinical management. Previous research has established that the MMRd subtype of EC presents pathohistological features that are different from the MMRp subtype. It has been demonstrated that these pathohistological characteristics, including elevated tumor grade, increased tumor size, a high number of tumor-infiltrating lymphocytes, extensive myometrial invasion, and the presence of lymphovascular space invasion (LVSI), are associated with MMRd status [16, 17]. Furthermore, these pathological tissue changes of MMRd subtype are associated with alterations in glucose metabolism and blood perfusion.

[^18^F]fluorodeoxyglucose ([^18^F]FDG) positron emission tomography (PET) is a common examination for evaluating the glucose metabolism of tumors. Sudo et al. [18] demonstrated that total lesion glycolysis (TLG) and metabolic tumor volume (MTV) of [^18^F]FDG PET were diagnostic indicators of LVSI in EC. Additionally, the maximum standard uptake value (SUV_max_) of [^18^F]FDG was higher in EC with a higher grade, a deeper myometrial invasion, and a larger tumor volume [19]. Dynamic contrast-enhanced magnetic resonance imaging (DCE-MRI) based on tracer kinetic models provides quantitative parameters to measure the blood perfusion of EC [20–22]. Transfer constant (K_trans_) and extravascular extracellular volume (V_e_) values derived from DCE-MRI were associated with the differentiation grade of EC [23]. Morever, K_trans,_ V_e,_ and efflux rate (K_ep_) were correlated with deep myometrial invasion and proliferation of EC [20, 22]. Therefore, we hypothesized that combining glucose metabolism and blood flow perfusion imaging could provide potential value for non-invasive identification of MMRd in EC.

Hybrid [^18^F]FDG PET/MRI could provide simultaneous glucose metabolism and blood perfusion assessments of EC. Some studies have demonstrated that hybrid PET/MRI performs well in evaluating myometrial invasion, stage, and prognosis of EC [24–28]. Nevertheless, there is a paucity of comprehensive investigation into the utility of PET/MR in forecasting the molecular subtype of EC. The present study is therefore designed to explore the potential association between MMRd and concomitant alterations in metabolic and blood perfusion processes. Furthermore, the identification of a specific pattern of metabolic and perfusion abnormalities could serve as an imaging biomarker for the guidance of immune checkpoint therapy decisions and the assessment of prognosis in early-stage EC.

## Methods

### Subjects

This prospective research study recruited patients who had experienced postmenopausal bleeding (PMB) and were initially diagnosed with EC on ultrasound between August 2021 and October 2023. The initial diagnosis of EC by ultrasound was performed by two ultrasound physicians with over 5 years of experience in gynecologic oncology, following the consensus statement by the International Endometrial Tumor Analysis group [29]. The exclusion criteria for this research were as follows: (1) PET/MR scan contraindications; (2) biopsy by hysteroscopy confirming non-malignant endometrial cell; and (3) postoperative pathology confirmed advanced (stage III and IV) EC by the 2009 version of the International Federation of Gynecology and Obstetrics (FIGO) staging criteria. This study was approved by the institutional ethics committee of Xuanwu hospital, and informed consent was obtained from all patients.

### PET/DCE-MRI protocol

All PET/DCE-MRI scans of patients were performed on a hybrid 3.0-T PET/MR (Signa, GE Healthcare) scanner with a 32-channel body coil. Following a minimum of 4 h of fasting to ensure a low blood glucose level (4–7 mmol/L), participants received an administration of [^18^F]FDG (14 MBq·min·bed^−1^·kg^−1^). The acquisition time interval for whole-body PET/MRI was 45–105 min following the intravenous injection of [^18^F]FDG. PET were scanned in the 3D list mode for 20 min (an acquisition time of 4-minutes per bed position and 5 bed positions) from above the head to pelvic. PET images were reconstructed by the three-dimensional (3D) iterative ordered-subset expectation maximization algorithm (3 iterations, 28 subsets, 2.78 mm slice thickness, and 192 × 192 image matrix) with time-of-flight information. Simultaneous whole-body MRI and pelvic DCE-MRI scans were performed for a total of approximately 60 min while PET data were acquired at each bed position. A two-point Dixon MRI sequence was scanned in per bed position for attenuation correction of the corresponding PET data. The detailed whole-body PET/MRI protocol is shown in Supplementary Fig. 1.

T1-weighted fast spin-echo (FSE) and T2-weighted propeller sequence were performed in per bed position. The pelvic MRI included conventional diagnostic MRI and DCE-MRI sequences. First, a large field of view (FOV) axial T1-weighted FSE sequence and a large FOV T2-weighted FSE were scanned. Then, high-resolution small FOV T2-weighted fat suppression propeller sequence (T2 fs) propeller sequence of axial, coronal, and sagittal images were scanned. Subsequently, pelvic DCE-MRI were obtained using a 3D Dixon sequence with the administration of gadolinium-based contrast (Gd-DTPA; 0.1 mmol/kg, 4.0 mL/s; Bayer Pharmaceutical, Germany) during 9 to 360 s (40 frames at 9 s per frame) [20]. Finally, single-phase sagittal, axial, and coronal T1 contrast enhanced (T1 CE) images were scanned. The detailed pelvic DCE-MRI parameters are described in Supplementary Table S1.

### Glucose metabolism and blood flow perfusion

**Fig. 1:**
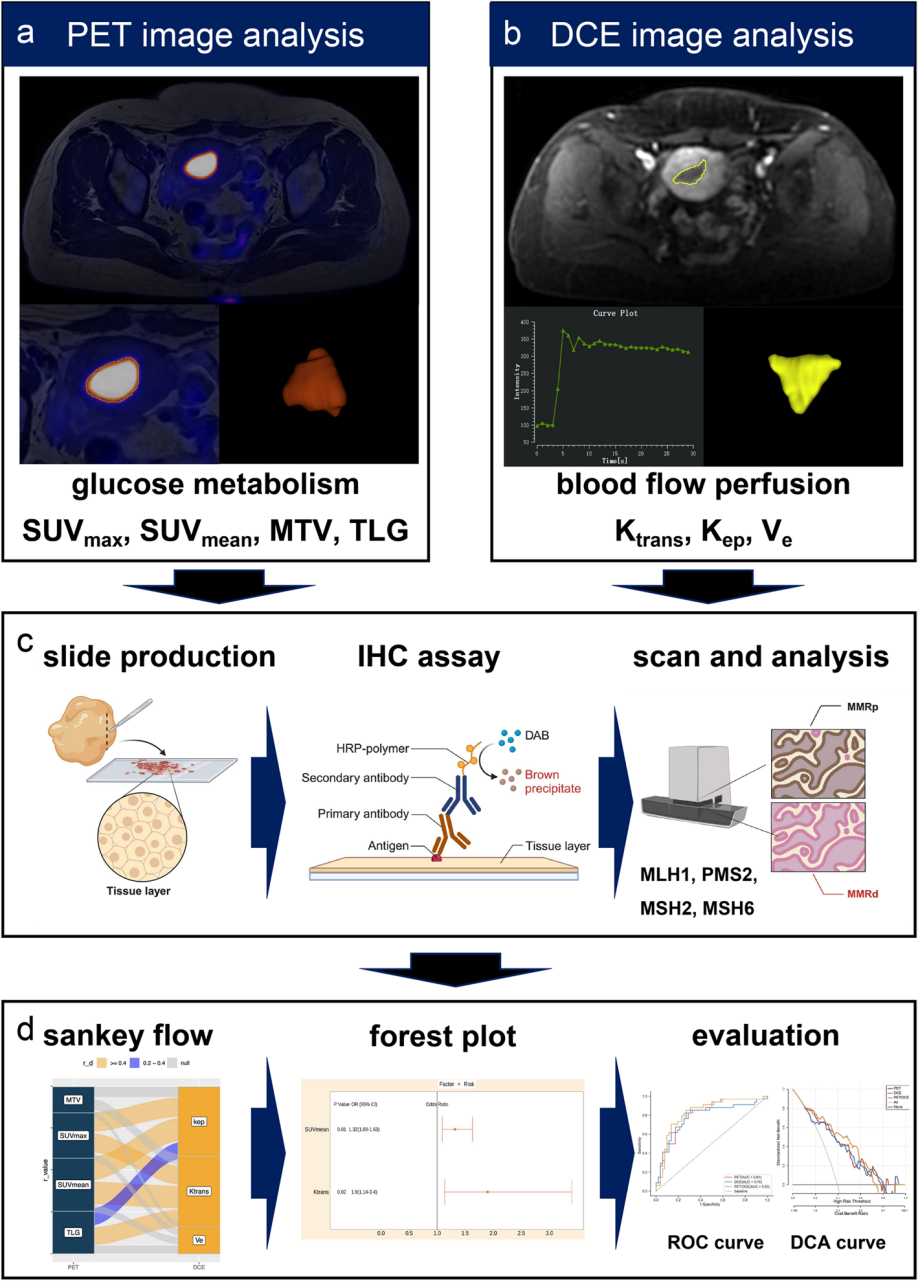
Flowchart of data analysis. **a** SUV_max_, SUV_mean_, MTV, and TLG were measured semi-automatically at the threshold of 40% on PET images. **b** K_trans_, K_ep_, and V_e_ were generated by standard Tofts pharmacokinetic model. **c** IHC staining and analysis of mismatch repair deficiency proteins. **d** Co-evolution analysis of glucose metabolism and blood flow perfusion in endometrial cancer. *SUV*_*max*_ maximum standardized uptake value, *SUV*_*mean*_ mean standardized uptake value, *MTV* metabolic tumor volume, *TLG* total lesion glycolysis, *K*_*trans*_ transfer constant, *K*_*ep*_ efflux rate, *V*_*e*_ extravascular extracellular volume, *IHC* Immunohistochemistry

All quantitative analyses of pelvic PET/MRI were processed with an AW workstation (version 4.7, GE Healthcare) by a nuclear medicine physician and a radiologist who both had 5 years of experience in pelvic tumor diagnosis blinded to clinical and pathological data. The flowchart of data analysis in this study is demonstrated in Fig. 1. SUV_max_, mean standardized uptake value (SUV_mean_), MTV, and TLG (MTV*SUV_mean_) were calculated by setting the 3D ellipsoidal region of interest (ROI) semi-automatically at the threshold of 40% on PET images (Fig. 1a). Quantitative analyses of DCE-MRI were processed by the GenIQ software within the AW workstation. The blood flow perfusion quantitative maps (K_trans_, K_ep_, and V_e_) were generated by the standard Tofts pharmacokinetic model with the individual arterial input function (AIF) obtained from the iliac artery [20]. Then, the maps of K_trans_, K_ep_, and V_e_ were loaded into the 3D slicer software (https://www.slicer.org). 3D ROI was manually delineated on the axial T1 CE images, which excluded obvious areas of necrosis, cystic degeneration, and hemorrhage (Fig. 1b) [22, 30]. Then, the same ROI was copied to parametric maps for calculating K_trans_, K_ep_, and V_e_.

### IHC analysis

The IHC staining and analysis of MMRd were performed by one pathologist with more than ten years of experience in oncology pathological diagnosis using the EnVision+/horseradish peroxidase method. Figure 1c showed the steps of IHC analysis for MMR protein. Formalin fixed paraffin embedded EC tumor tissues were first treated with xylene to remove the paraffin. Then, the tumor slides were blocked with 10% goat serum following sequentially incubated with primary antibodies and second antibody. The following monoclonal antibodies were used for four MMRd proteins (MLH1, PMS2, MSH2, and MSH6) IHC staining: MLH1 (mouse monoclonal, 1:100, ZSGB-BIO, Beijing, China), PMS2 (rabbit monoclonal, 1:100, ZSGB-BIO, Beijing, China), MSH2 (1:100, ZSGB-BIO, Beijing, China), and MSH6 (rabbit monoclonal, 1:100, ZSGB-BIO, Beijing, China). Then the polymer horseradish peroxidase detection system using diaminobenzidine (Polink-1HRP Broad Spectrum DAB Detection Kit, Golden Bridge International, USA) was used for incubating tumor slides. All IHC slides were scanned at 400×magnification using Leica Biosystems (Leica Aperio AT Turbo, USA). The MMRd of EC was defined by a complete loss of nuclear expression in carcinoma cells of at least one of the MMR proteins (MLH1, MSH2, MSH6, and PMS2) with stromal and/or lymphocytic cells as internal controls recommended by the practical guidance [31].

#### Statistical analysis

All continuous data in this study were presented as the mean ± standard error (SD). The independent sample t test was used for comparing the PET and DCE-MRI parameters between the MMRd and MMRp groups. The synchronous variation of glucose metabolism parameters and blood perfusion parameters of primary tumors was analyzed using the Sankey diagram based on Spearman’s correlation test (Fig. 1d). Univariate and multivariate logistic regression were used to establish digital biomarkers combining the PET and DCE-MRI parameters for diagnosing the MMRd of early-stage EC (Fig. 1d). The evaluation and comparison of digital biomarkers derived from multimodal PET/DCE-MRI for diagnosing MMRd were performed by the receiver operating characteristic (ROC) curve, decision curve analysis (DCA), and the net reclassification index (NRI). All statistical analyses were processed by the R software (version 4.3.1, R Foundation for Statistical Computing, Austria). The statistical and highly statistical significance were defined as *P* values < 0.05 and 0.01, respectively.

## Results

### Clinicopathologic information of enrolled participants

**Fig. 2:**
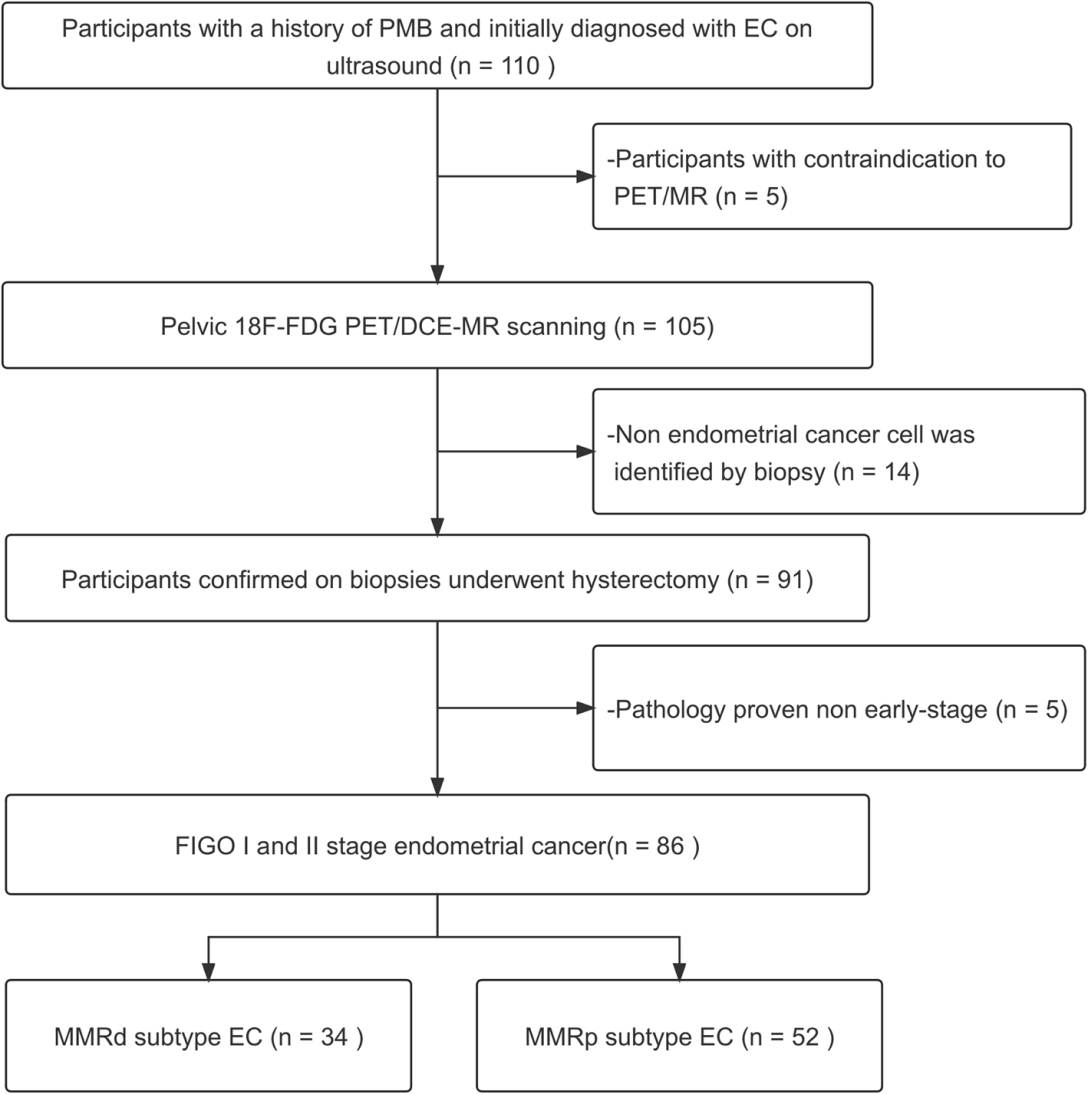
Inclusion and exclusion results of this study. *PMB* postmenopausal bleeding, *FIGO* International Federation of Gynecology and Obstetrics, *MMRd* mismatch repair deficiency, *MMRp* mismatch repair proficiency

A total of 110 participants with a history of PMB and initially diagnosed with primary EC were consecutively recruited (Fig. 2). 105 participants underwent pelvic [^18^F]FDG PET/DCE-MRI scanning after excluding 5 participants with contraindications to PET/MR scanning. Finally, 86 participants with early-stage EC were enrolled in this study following the hysterectomy.

The clinicopathologic characteristics of the enrolled participants with early-stage EC are demonstrated in Table 1. There were 34 cases with MMRd and 52 cases with MMRp in all EC cases. Histologic subtypes of tumors included endometrioid carcinoma (95.35%), serous adenocarcinoma (3.49%), and clear cell carcinoma (1.16%). The majority of the EC cases included in this study were of FIGO I stage (IA and IB, 91.87%), superficial myometrial invasion (MI ≤ 1/2, 66.28%), and low-grade differentiation endometrioid carcinoma (grades 1 and 2, 84.88%).

**Table 1:** Demographics of patients

Demographics	All patients (*n* = 86)
Age (years old), mean ± SD	58.92 ± 10.13
CA-125 (U/ml), mean ± SD	25.88 ± 31.06
FIGO stage, *n* (%)	
IA	52 (60.47%)
IB	27 (31.40%)
II	7 (8.13%)
Histologic subtype, *n* (%)	
Endometrioid carcinoma, grade 1	22 (25.58%)
Endometrioid carcinoma, grade 2	51 (59.30%)
Endometrioid carcinoma, grade 3	9 (10.47%)
Serous adenocarcinoma	3 (3.49%)
Clear cell carcinoma	1 (1.16%)
MI, *n* (%)	
≤ 1/2	57 (66.28%)
> 1/2	29 (33.72%)
MMR, *n* (%)	
MMRp	52 (60.47%)
MMRd	34 (39.53%)

Data were described as the mean ± SD or *n* (%). *SD indicates* standard deviation, *FIGO* International Federation of Gynecology and Obstetrics, *MI* myometrial invasion, *MMRd* mismatch repair deficiency, *MMRp* mismatch repair proficiency

### Clinical and PET/DCE-MRI parameters

The clinical and PET/DCE-MRI parameters comparisons were described in Table 2. There was no significant difference in age and CA-125 level between the MMRd subtype and the MMRp subtype (*P* > 0.05). The SUV_max_ (*P* < 0.001), SUV_mean_ (*P* < 0.001), MTV (*P* = 0.002), and TLG (*P* = 0.004) of 34 cases with the MMRd subtype were significantly higher than those of 52 cases with MMRp subtype. And K_trans_ (*P* < 0.001) and K_ep_ (*P* = 0.005) were also higher in the MMRd subtype than in the MMRp subtype. But the difference in V_e_ (*P* = 0.302) between the MMRd subtype and the MMRp subtype was not found.

**Table 2:** Clinical and PET/DCE-MRI parameters

variables	MMRp(*n* = 52)	MMRd (*n* = 34)	t	*P* value
Age, mean ± SD	59.65 ± 10.38	57.79 ± 9.45	0.83	0.408
CA-125, mean ± SD	21.79 ± 19.49	32.12 ± 42.03	−1.52	0.133
SUV_max_, mean ± SD	9.58 ± 9.17	18.73 ± 9.70	−4.37	< 0.001
SUV_mean_, mean ± SD	4.72 ± 2.50	7.55 ± 2.76	−4.86	< 0.001
MTV, mean ± SD	7.57 ± 9.75	19.64 ± 19.18	−3.35	0.002
TLG, mean ± SD	46.83 ± 95.58	174.73 ± 226.02	−3.08	0.004
Volume_MR, mean ± SD	19.11 ± 92.44	26.67 ± 60.20	−0.42	0.678
K_trans_, mean ± SD	0.88 ± 0.93	1.92 ± 1.18	−4.50	< 0.001
V_e_, mean ± SD	0.42 ± 0.21	0.47 ± 0.23	−1.04	0.302
k_ep_, mean ± SD	2.17 ± 2.02	6.81 ± 8.74	−3.00	0.005

*MMRd indicates* mismatch repair deficiency, *MMRp*, mismatch repair proficiency, *SD* standard deviation, *SUV*_*max*_, maximum standardized uptake value, *SUV*_*mean*_, mean standardized uptake value, *MTV* metabolic tumor volume, *TLG* total lesion glycolysis, *K*_*trans*_, transfer constant, *K*_*ep*_, efflux rate, *V*_*e*_, extravascular extracellular volume

### Synchronous variation of glucose metabolism and blood perfusion

**Fig. 3:**
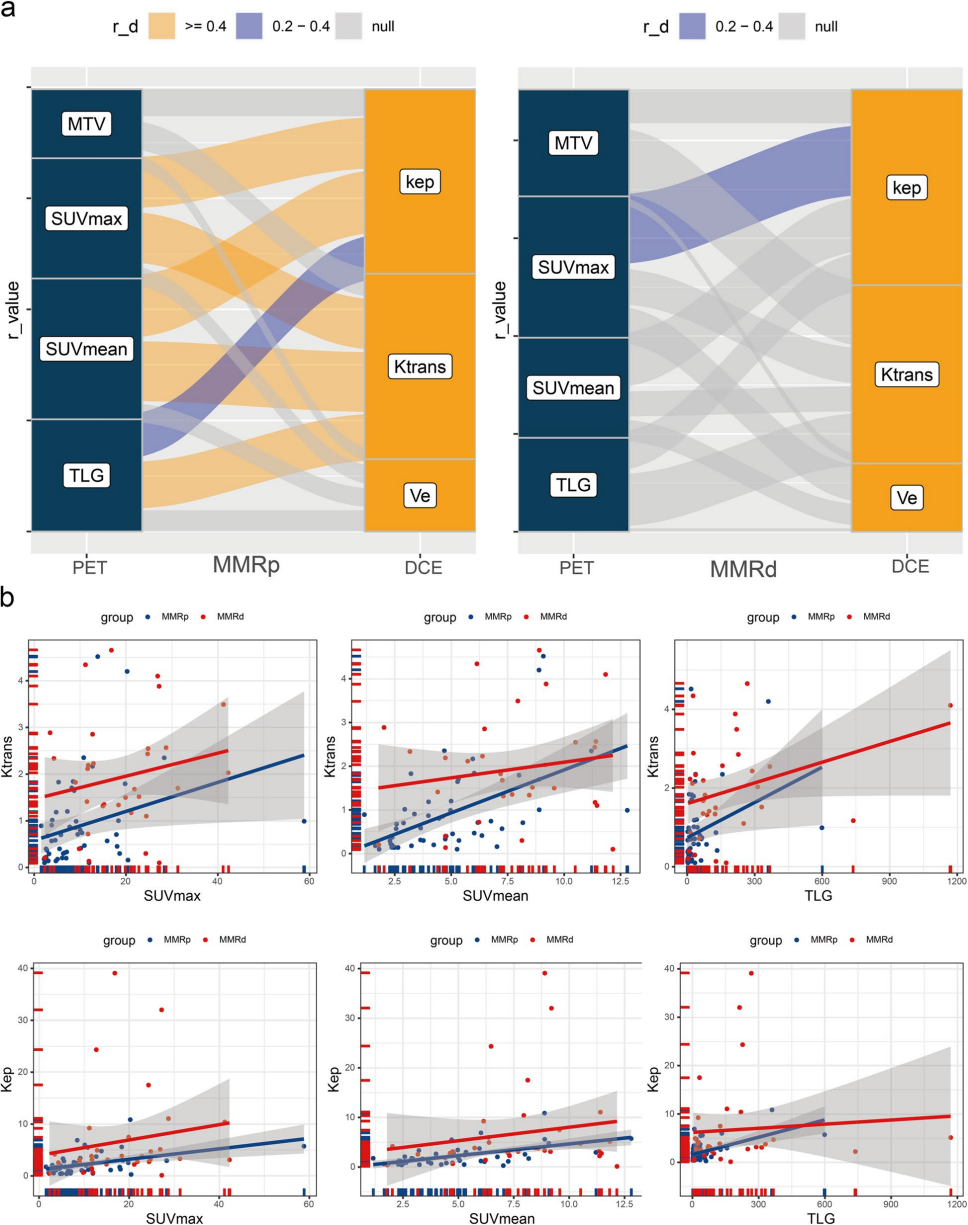
Co-reactivity of glucose metabolism and blood flow perfusion in endometrial cancer. **a** The Sankey diagram showed the correlation between glucose metabolism and blood flow perfusion in MMRd and MMRp subtypes. **b** Scatterplot of correlation between glucose metabolism and blood flow perfusion in MMRd and MMRp subtypes. *MMRd* mismatch repair deficiency, *MMRp* mismatch repair proficiency

**Fig. 4:**
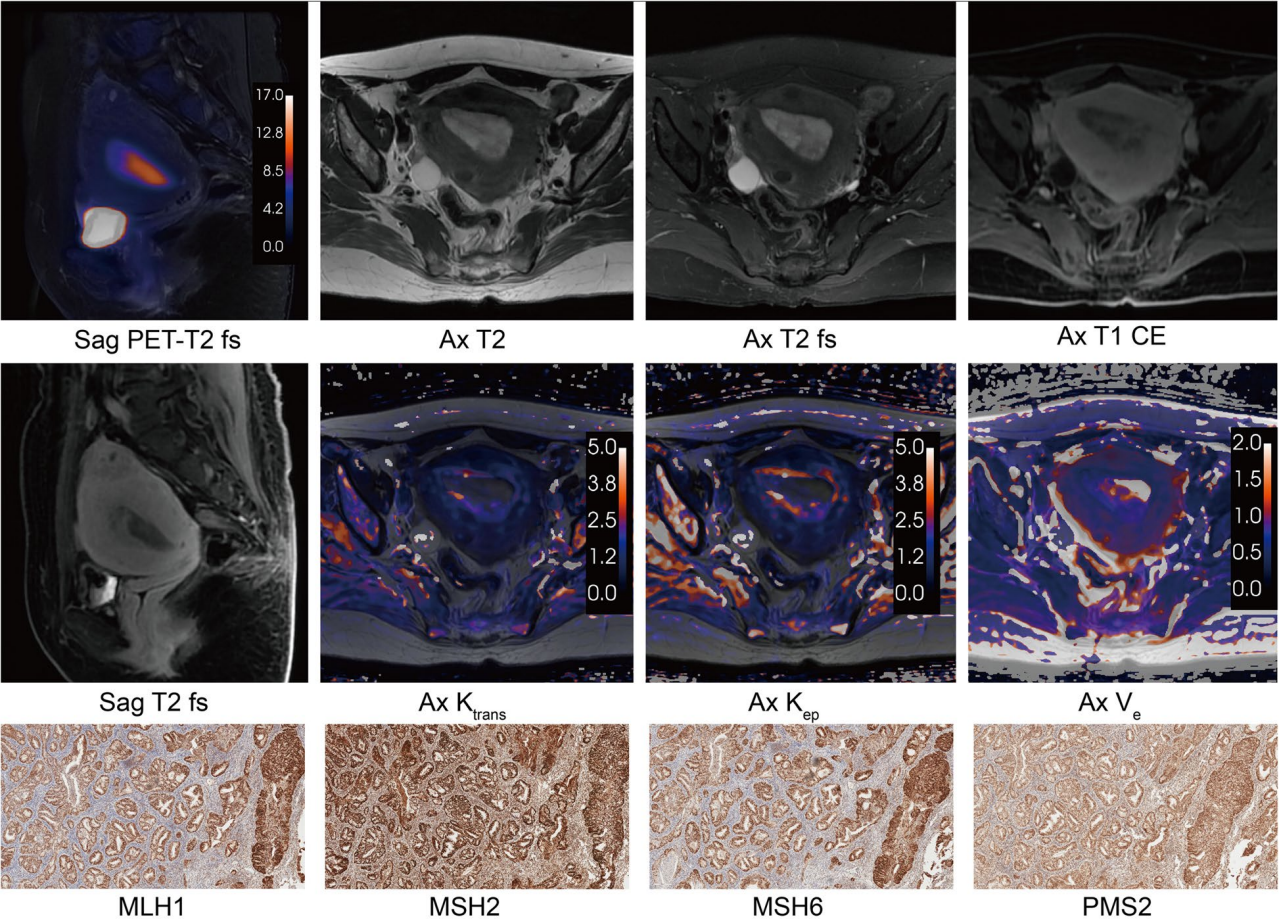
The PET/DCE-MRI images and immunohistochemistry of a 51-year-old female with MMRp endometrioid carcinoma (FIGO IA stage, grade 2). An intrauterine mass showed glucose moderate hypermetabolism (SUV_max_ = 12.71, SUV_mean_ = 6.48, MTV = 35.08, TLG = 227.16) and hypo blood flow perfusion (K_trans_ = 0.72, K_ep_ = 0.25, V_e_ = 2.88). **a** Sagittal PET and T2 fs fused image. **b** Axial T2-weighted image. **c** Axial T2 fs image. **d** Axial T1 CE image. **e** Sagittal T1 CE image. **f** Axial K_trans_ map. **g** Axial K_ep_ map. **h** Axial V_e_ map. **i** MLH1 protein immunohistochemical staining (× 100). **j** MSH2 protein immunohistochemical staining (× 100). **k** MSH6 protein immunohistochemical staining (× 100). **l** PMS2 protein immunohistochemical staining (× 100). *T2 fs* T2-weighted fat suppression, *T1 CE* T1-weighted contrast enhanced, *SUV*_*max*_ maximum standardized uptake value, *SUV*_*mean*_ mean standardized uptake value, *MTV* metabolic tumor volume, *TLG* total lesion glycolysis, *K*_*trans*_ transfer constant, *K*_*ep*_ efflux rate, *V*_*e*_ extravascular extracellular volume

**Fig. 5:**
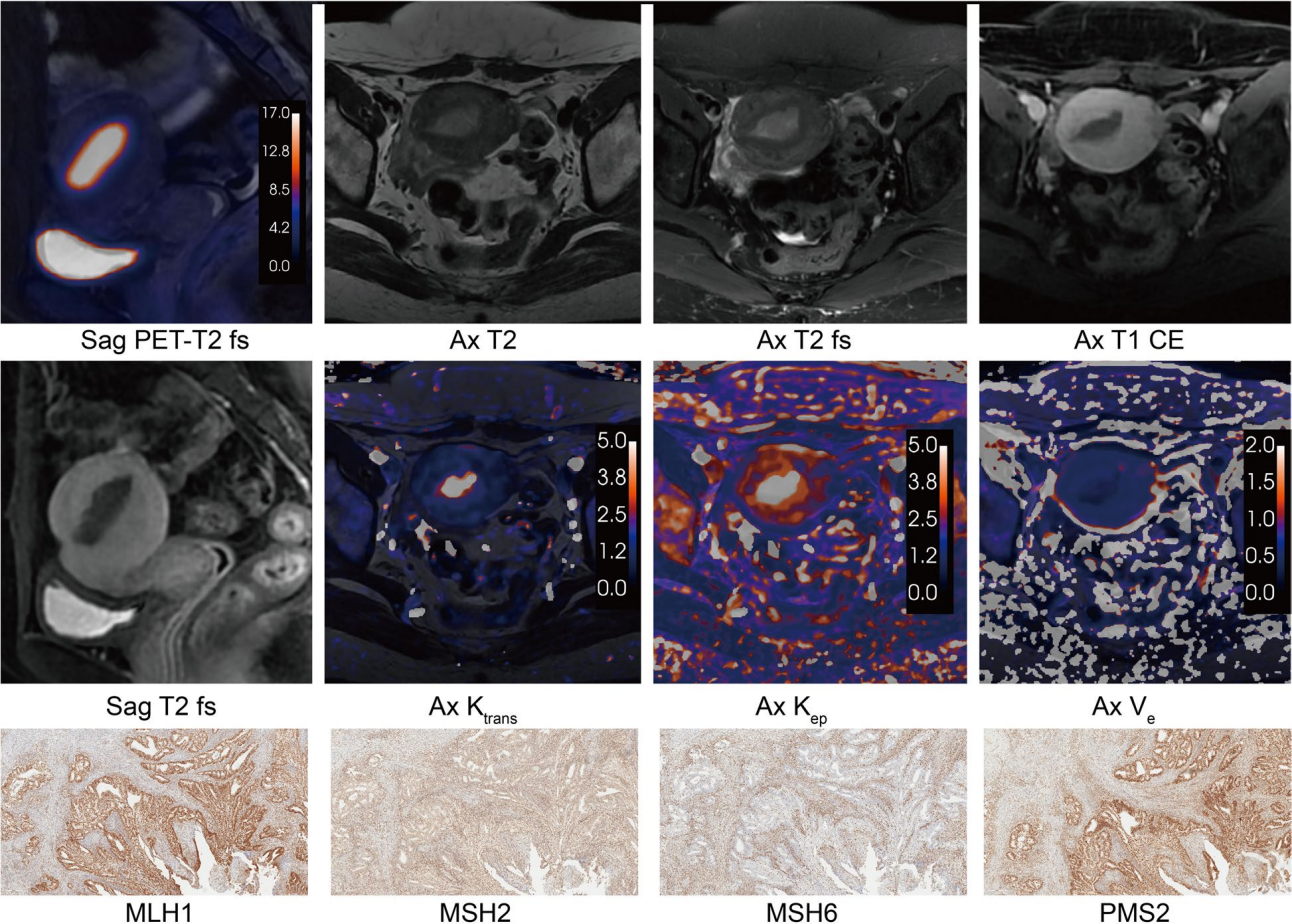
The PET/DCE-MRI images and immunohistochemistry of a 48-year-old female with MMRd endometrioid carcinoma (FIGO IA stage, grade 1). An intrauterine mass showed significant glucose hypermetabolism (SUV_max_ = 28.73, SUV_mean_ = 11.40, MTV = 13.71, TLG = 156.39) and hyper blood flow perfusion (K_trans_ = 4.2, K_ep_ = 11.06, V_e_ = 0.37). **a** Sagittal PET and T2 fs fused image. **b** Axial T2-weighted image. **c** Axial T2 fs image. **d** Axial T1 CE image. **e** Sagittal T1 CE image. **f **Axial K_trans_ map. **g** Axial K_ep_ map. **h** Axial V_e_ map. **i** MLH1 protein immunohistochemical staining (× 100). **j** MSH2 protein immunohistochemical staining (× 100). **k** MSH6 protein immunohistochemical staining (× 100). **l** PMS2 protein immunohistochemical staining (× 100). *T2 fs* T2-weighted fat suppression, *T1 CE* T1-weighted contrast enhanced, *SUV*_*max*_ maximum standardized uptake value, *SUV*_*mean*_ mean standardized uptake value, *MTV* metabolic tumor volume, *TLG* total lesion glycolysis, *K*_*trans*_ transfer constant, *K*_*ep*_ efflux rate, *V*_*e*_ extravascular extracellular volume

The different relationships between glucose metabolism and blood perfusion were demonstrated in the MMRd and MMRp subgroups of early-stage EC (Fig. 3a). In the MMRp subgroup, K_trans_ was correlated with SUV_max_ (*r* = 0.45, *P* < 0.001), SUV_mean_ (*r* = 0.53, *P* < 0.001), and TLG (*r* = 0.42, *P* = 0.002). And K_ep_ was simultaneously correlated with SUV_max_ (*r* = 0.46, *P* < 0.001), SUV_mean_ (*r* = 0.56, *P* < 0.001), and TLG (*r* = 0.40, *P* = 0.003). In the MMRd group, SUV_max_ was correlated with K_ep_ (*r* = 0.36; *P* = 0.039). There was no significant correlation between MTV and all DCE-MRI parameters in both subgroups (*P* > 0.05). And V_e_ was not associated with all PET parameters in both subgroups (*P* > 0.05). MMRd subtype cases were more distributed in locations with extreme both high metabolism and perfusion, and the correlation between blood perfusion and glucose metabolism was not significant (Fig. 3b). But in the MMRp subgroup, blood perfusion tended to increase significantly and consistently with increasing glucose metabolism (Fig. 3b). The MMRp subtype and MMRd subtype PET/DCE-MRI and IHC characteristics were demonstrated in Figs. 4 and 5, respectively.

### Multimodal imaging biomarker establishment

Univariate logistic regression analysis demonstrated that SUV_max_ (*P* < 0.001), SUV_mean_ (*P* < 0.001), MTV (*P* < 0.001), TLG (*P* = 0.004), K_trans_ (*P* < 0.001) and K_ep_ (*P* = 0.005) were positively associated with MMRd in early-stage EC (Table 3). For PET modal digital biomarker establishment, TLG (odds ratio [OR] = 0.99; 95% confidence interval [CI] = 0.98–1.00, *p* = 0.100), MTV (OR = 1.13; 95% CI = 1.01–1.28, *P* = 0.030), and SUV_mean_ (OR = 1.53; 95% CI = 1.18–2.03, *P* < 0.001) were selected by multivariate logistic regression analysis (Supplementary Fig. 2a). K_ep_ (OR = 1.17; 95% CI = 0.99–1.5, *P* = 0.110) and K_trans_ (OR = 1.86; 95% CI = 1.02–3.5, *P* = 0.040) were independent risk factors for calculating the DCE-MRI modal digital biomarker (Supplementary Fig. 2b). SUV_mean_ (OR = 1.32; 95% CI = 1.09–1.63, *P* = 0.006) and K_trans_ (OR = 1.90; 95% CI = 1.14–3.40; *P* = 0.021) were independent risk factors for calculating the PET/DCE modal digital biomarker (Supplementary Fig. 2c; Table 3).

**Table 3:** Logistic regression analysis of PET/DCE-MRI

Variables	Univariate analysis			Multivariate analysis		
	OR	95%CI	*P* value	OR	95%CI	*P* value
SUV_max_	1.11	1.05–1.18	< 0.001	-	-	-
SUV_mean_	1.45	1.20–1.75	< 0.001	1.32	1.09–1.63	0.006
MTV	1.08	1.03–1.13	< 0.001	-	-	-
TLG	1.01	1.00–1.01	0.004	-	-	-
K_trans_	2.47	1.48–4.11	< 0.001	1.90	1.14–3.40	0.021
V_e_	2.39	0.31–18.77	0.406	-	-	-
k_ep_	1.35	1.10–1.65	0.005	-	-	-

*CI *indicates confidence interval,* SUV*_*max*_, maximum standardized uptake value, *SUV*_*mean*_, mean standardized uptake value, *MTV* metabolic tumor volume, *TLG* total lesion glycolysis, *K*_*trans*_, transfer constant, *K*_*ep*_, efflux rate, *V*_*e*_, extravascular extracellular volume

### Diagnostic performance

**Fig. 6:**
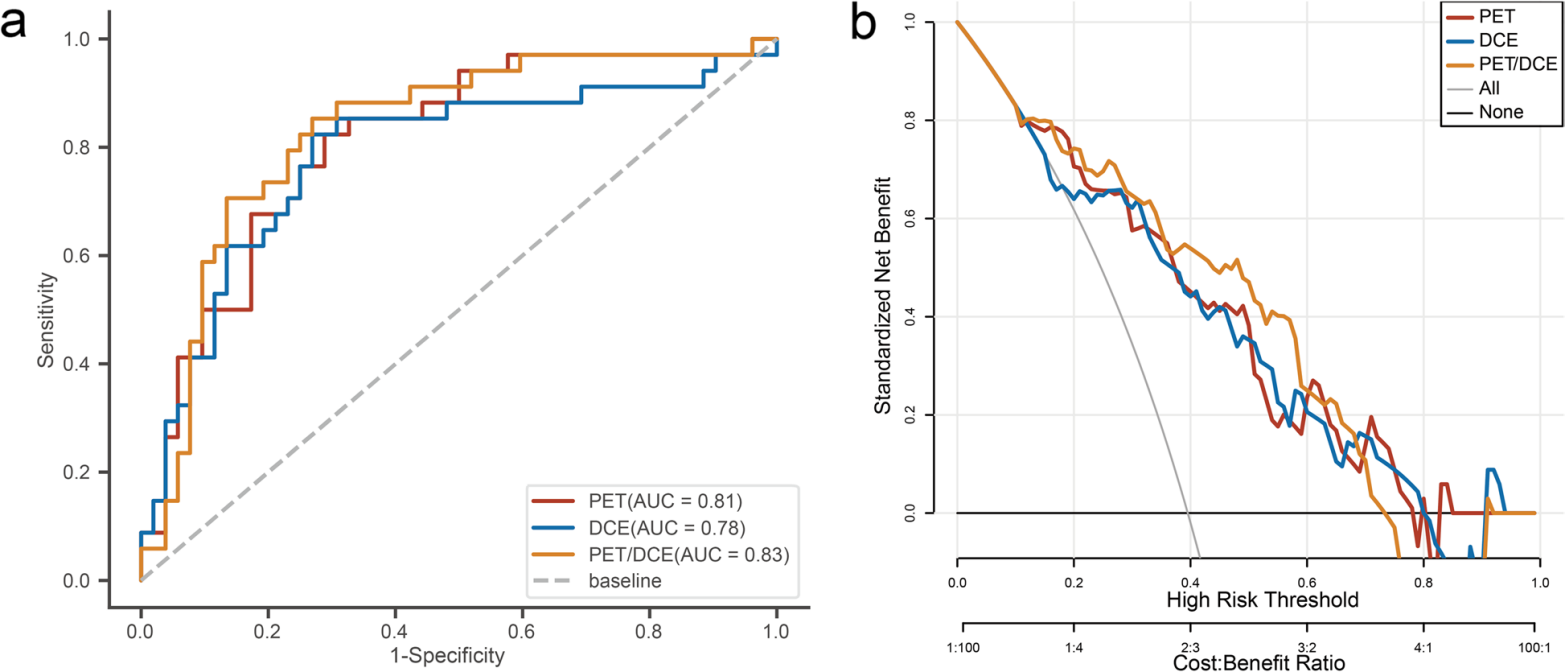
Evaluation of digital biomarker integrating PET and DCE-MRI. **a** ROC curves of PET/DCE-MRI digital biomarkers for identifying MMRd in early-stage EC. **b** DCA curves of PET/DCE-MRI digital biomarkers for identifying MMRd in early-stage EC. *ROC* receiver operating characteristic, *DCA* decision curve analysis, *MMRd* mismatch repair deficiency, *EC* endometrial cancer

The PET/DCE modal digital biomarker performed best in differentiating MMRd from MMRp in early-stage EC (area under the ROC curve [AUC] = 0.83, accuracy = 0.78, sensitivity = 0.85, and specificity = 0.73) (Fig. 6a). And the PET/DCE-MRI modal digital biomarker provided additional diagnostic effectiveness compared to single-modality imaging for the diagnosis of MMRd in early-stage EC (Fig. 6b). The detailed performance of PET and DCE digital biomarkers in diagnosing MMRd is described in Table 4. The NRI analysis demonstrated that the PET/DCE-MRI digital biomarker improved the accuracy of this diagnosis for MMRd by 4% and 13% compared to PET modal and DCE modal, respectively (Supplementary Table S2).

**Table 4:** ROC evaluation of PET/DCE-MRI

Image modality	AUC (95% CI)	Sensitivity	Specificity	Accuracy
PET	0.81 (0.72–0.90)	0.82	0.71	0.76
DCE	0.78 (0.68–0.89)	0.82	0.73	0.77
PET/DCE	0.83 (0.74–0.92)	0.85	0.73	0.78

*AUC *indicates area under curve, *CI* confidence interval

## Discussion

The importance of MMRd testing in early-stage EC has been noted by the European Society of Medical Oncology (ESMO), the European Society of Gynaecological Oncology (ESGO), and the European SocieTy for Radiotherapy and Oncology (ESTRO) guidelines. This study set out to discover the association of simultaneous change patterns in glucose metabolism and blood perfusion with MMRd in early-stage EC and establish a digital biomarker based on the specific co-reactivity of glucose metabolism and blood perfusion for non-invasive identification of MMRd to guide adjuvant treatment decisions and evaluate the prognosis of early-stage EC. The results of this study indicated that the EC patients with MMRd had a simultaneous increase in glucose metabolism and blood perfusion compared to those with the MMRp subtype. One unanticipated result was that the correlation of glucose metabolism on PET images and blood perfusion on DCE-MRI was significantly different between MMRp and MMRd subtypes. And a digital biomarker integrating metabolism and blood perfusion based on PET/DCE-MRI performed best in demonstrating MMRd in early-stage EC among multimodal imaging.

There are few studies evaluating MMRd in EC based on MRI or PET images. Minamiguchi et al. found that the tumor location of MMRd EC significantly presented in the lower uterine site [32]. However, visual determination of tumor location on an MRI was difficult and lacked quantitative parameter analysis for clinical diagnosis because some endometrial cancer tumors show diffuse infiltrative growth or only thickened endothelium. Li et al. [33] showed that the amide proton transfer-weighted (APTw) value was significantly higher in the MMRd group than in the MMRp group, and they suggested that this was due to the ability of APTw to reflect the tumor microenvironment. Our results that the SUV_max,_ SUV_mean_, MTV, and TLG of the MMRd subtype were significantly higher than those of the MMRp subtype also supported their findings. The tumor immune microenvironment (TME) of MMRd subtype EC was more sophisticated than that of MMRp subtype EC and had more lymphocyte infiltration and more PD-L1/PD-1 protein expression [6, 34]. Macrophages and T cells in the TME related to MMRd with higher glucose uptake have been confirmed [35]. Thus, the glucose metabolism parameters and APTw values increased in the MMRd subtype EC. Other studies similarly found that SUV_max_, MTV, TLG, and magnetization transfer ratio asymmetry (MTRasym = 3.5 ppm) were higher in PD-L1 positive non-small cell lung cancer (NSCLC) than in PD-L1 negative NSCLC [36]. This finding of our study was consistent with that of Sun et al. [37] who also demonstrated that higher SUV_max_ was observed in the MMRd subtype EC.

Furthermore, the higher K_trans_ and K_ep_ based on DCE-MRI in MMRd subtype EC revealed increased vascular permeability in the MMRd subtype compared to that of the MMRp subtype. K_trans_ and K_ep_ reflect the rate of contrast agent permeation from intravascular to tumor tissue and reverse permeation, respectively. A possible explanation for this might be that the MMRd subtype EC exhibited hypoxia and more neovascularization to facilitate the exchange of contrast agents in and out of the vasculature. The high expression of vascular endothelial growth factor (VEGF) in MMRd subtype supported this hypothesis [38, 39]. Previous studies have also found that high blood perfusion was present in the pathological tissue associated with MMRd. K_trans_ and K_ep_ of high-proliferation EC were significantly higher than those of low-proliferation EC, but V_e_ was not significantly different, which could confirm our results [20]. Since MMRd protein expression was associated with Ki-67 levels, the MMRd subtype showed high proliferation [40, 41]. In contrast, Ye et al. [22] discovered that high-risk EC exhibited significantly lower K_trans_ and K_ep_ compared to those of other EC. This rather contradictory result may be due to the fact that the definition of high-risk EC was not correlated with MMRd and different pharmacokinetic models were used in their study. The presence of LVSI significantly associated with MMRd was rare in the high-risk EC of their study.

The disparate correlation observed between glucose metabolism and blood perfusion across the MMRd and MMRp cohorts can be explained by the mediation of TME and interstitial fluid pressure. Since the TME of MMRp endometrial cancer is relatively homogeneous and lacks immune cell infiltration, glucose metabolism in tumors tissue with MMRp is dominated by tumor cell uptake [6, 42]. Increased proliferation of tumor promotes highly permeable neovascularization in the MMRp. Therefore, glucose metabolism parameters were correlated with blood perfusion in the MMRp group. However, increased heterogeneity of the TME due to immune cell infiltration is accompanied by increased interstitial fluid pressure in the MMRd subtype [43]. Increased interstitial fluid pressure caused by immune cell enrichment hinders contrast agent penetration from the vasculature into the tumor tissue [44]. This explains why glucose metabolism is not simply correlated with K_trans,_ despite the increased vascular permeability of MMRd. And the positive correlation between K_ep_ and SUV_max_ also proves it.

Therefore, a specific co-reactivity of glucose metabolism and blood perfusion could evaluate the MMRd in early-stage EC. The value of APTw (AUC = 0.78) in predicting MMRd in the previous study was the same as the DCE-MRI in our study (AUC = 0.78) [33]. And our study found that PET performed better for identifying MMRd than DCE-MRI (AUC, 0.81 vs. 0.78), and a combination of SUVmean and K_trans_ had a further improvement in the accuracy of identifying MMRd as compared to DCE-MRI (NRI = 13%). However, the addition of DCE-MRI to PET imaging did not result in a notable enhancement in the prediction of MMRd compared to PET alone (NRI = 4%). The findings of the present study align with those of previous research, which demonstrated that the EC of the MMRd subtype was infiltrated with immune cells that uptake greater quantities of [^18^F]FDG. Whereas increased perfusion in MMRd is a subsequent change due to immune cell infiltration, therefore DCE-MRI can further increase the accuracy of PET in identifying MMRd. The digital biomarker integrating metabolism and blood perfusion can provide additional identifying MMRd value for EC patients who underwent PET/MR examination.

However, we recognize that some limitations are present in this study. First, although this is a prospective PET/DCE-MRI study exploring the apparent alterations of glucose metabolism and blood flow perfusion mediated by MMRd in early-stage EC. But this study was performed at one PET/MR scanner with limited participants. Discrete multi-center PET/CT and MR scanners need to validate this finding in the future. Second, this study focused on the MMRd evaluation of participants with early-stage EC. Other molecular subtypes including P53 and POLE mutations can be further analyzed for risk stratification of all FIGO stage EC. Third, MMRd status was prevalent in other solid cancers, but the findings in this study lacks validation in other cancers. The generalizability of these findings maybe limited due to the specific TME in EC. Finally, PET/MR examinations are limited by equipment, examination costs and PET tracer, and therefore are not available for large-scale clinical applications in many regions. In the future, generative artificial intelligence techniques may be employed to generate PET images from MR images, thus facilitating further validation of our findings. Despite its limitations, the study certainly adds to our understanding of the co-reactivity of glucose metabolism and blood flow perfusion mediated by MMRd in EC.

## Conclusion

The co-activation pattern of glucose metabolism and blood flow perfusion can be used to reveal DNA MMRd in early-stage EC. Furthermore, the digital biomarker based on [^18^F]FDG PET/DCE-MRI has the potential value in non-invasively identifying MMRd for performing prognostic risk stratification in patients with early-stage EC.

## Supplementary Information

Supplementary Material 1.

## Data Availability

Data is provided within the manuscript or supplementary information files. Detailed original dates of this research are available from the corresponding author upon reasonable request.

## Abbreviations

DCE: Dynamic contrast-enhanced
EC: Endometrial cancer
FIGO: The International Federation of Gynecology and Obstetrics
[^18^F]FDG: [^18^F]fluorodeoxyglucose
IHC: Immunohistochemistry
K_ep_: Efflux rate
LVSI: Lymphovascular space invasion
MI: Myometrial invasion
MMRd: Mismatch repair deficiency
MMRp: Mismatch repair deficiency proficiency
MTV: Metabolic tumor volume
NRI: Net reclassification improvement
OR: Odds ratio
PMB: Postmenopausal bleeding
SUV: Standardized uptake value
TLG: Total lesion glycolysis
K_trans_: Transfer constant
T1 CE: T1 contrast enhanced
V_e_: Extravascular extracellular volume
