# Supplementary material for: Co-reactivity pattern of glucose metabolism and blood perfusion revealing DNA mismatch repair deficiency based on PET/DCE-MRI in endometrial cancer
Source: Cancer Imaging. 2024 Nov 25;24:161. doi: 10.1186/s40644-024-00805-5 (PMC11587675; doi:10.1186/s40644-024-00805-5)
Supplement: Supplementary file 1 — Supplementary Material 1. [file 40644_2024_805_MOESM1_ESM.docx]

**Supplementary materials**

**Supplementary Fig. 1.** Whole-body PET/MRI scanning protocol.

**Supplementary Fig. 2.** Forestplots of PET/DCE-MRI parameters for identifying MMRd.

**Supplementary Table S1** Detailed pelvic MRI parameters.

**Supplementary Table S2** NRI analysis for PET/DCE-MRI.

**
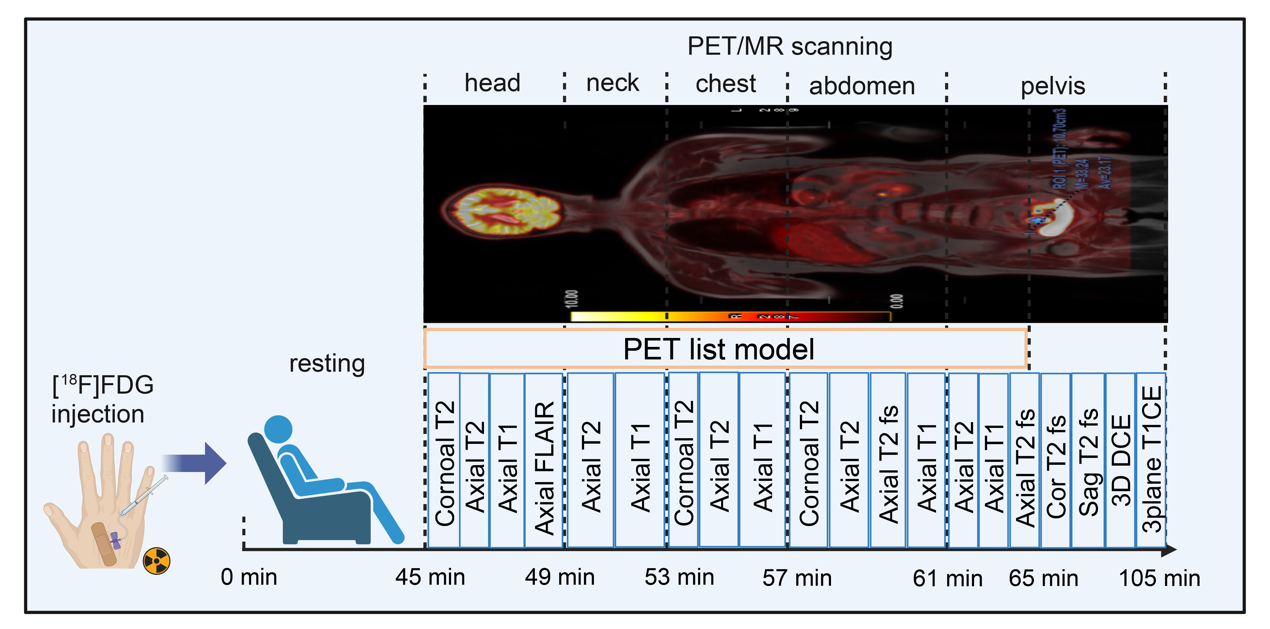
**

**Supplementary Fig. 1.** Whole-body PET/MRI scanning protocol. *DCE,* dynamic contrast-enhanced; *fs,* fat suppression.

**Supplementary Table S1** Detailed pelvic MRI parameters.

| **Parameter** | **Axial**  **T1** | **Axial**  **T2** | **Axial**  **T2 fs** | **Coronal**  **T2 fs** | **Sagittal**  **T2 fs** | **DCE** | **3 plane**  **T1 CE** |
| --- | --- | --- | --- | --- | --- | --- | --- |
| Sequence | FSE | FSE | FSE PROPELLER | FSE PROPELLER | FSE PROPELLER | 3D Dixon  GRE | 3D Dixon  GRE |
| TE (ms) | 10 | 70 | 75 | 69.08 | 69 | 1.0 | 1.79 |
| TR (ms) | 526 | 3900 | 6000 | 3135 | 4271 | 2.5 | 4.97 |
| FOV (cm) | 44 | 44 | 26 | 26 | 28 | 38 | 40 |
| Matrix size | 512×512 | 512×512 | 512×512 | 512×512 | 512×512 | 512×512 | 512×512 |
| Thickness (mm) | 4 | 4 | 4 | 4 | 4 | 4 | 4 |
| Pixel spacing (mm) | 0.85/0.85 | 0.85/0.85 | 0.50/0.50 | 0.50/0.50 | 0.54/0.54 | 0.74/0.74 | 0.78/0.78 |
| NEX | 1 | 2 | 4 | 2 | 2 | 1 | 1 |

*DCE,* dynamic contrast-enhanced; *fs,* fat suppression; *FOV,* field of view; *GRE,* gradient recolled echo; *NEX,* number of excitations; *PROPELLER*, periodically rotated overlapping parallel lines with enhanced reconstruction; *TE*, echo time; *TR,* repetition time.


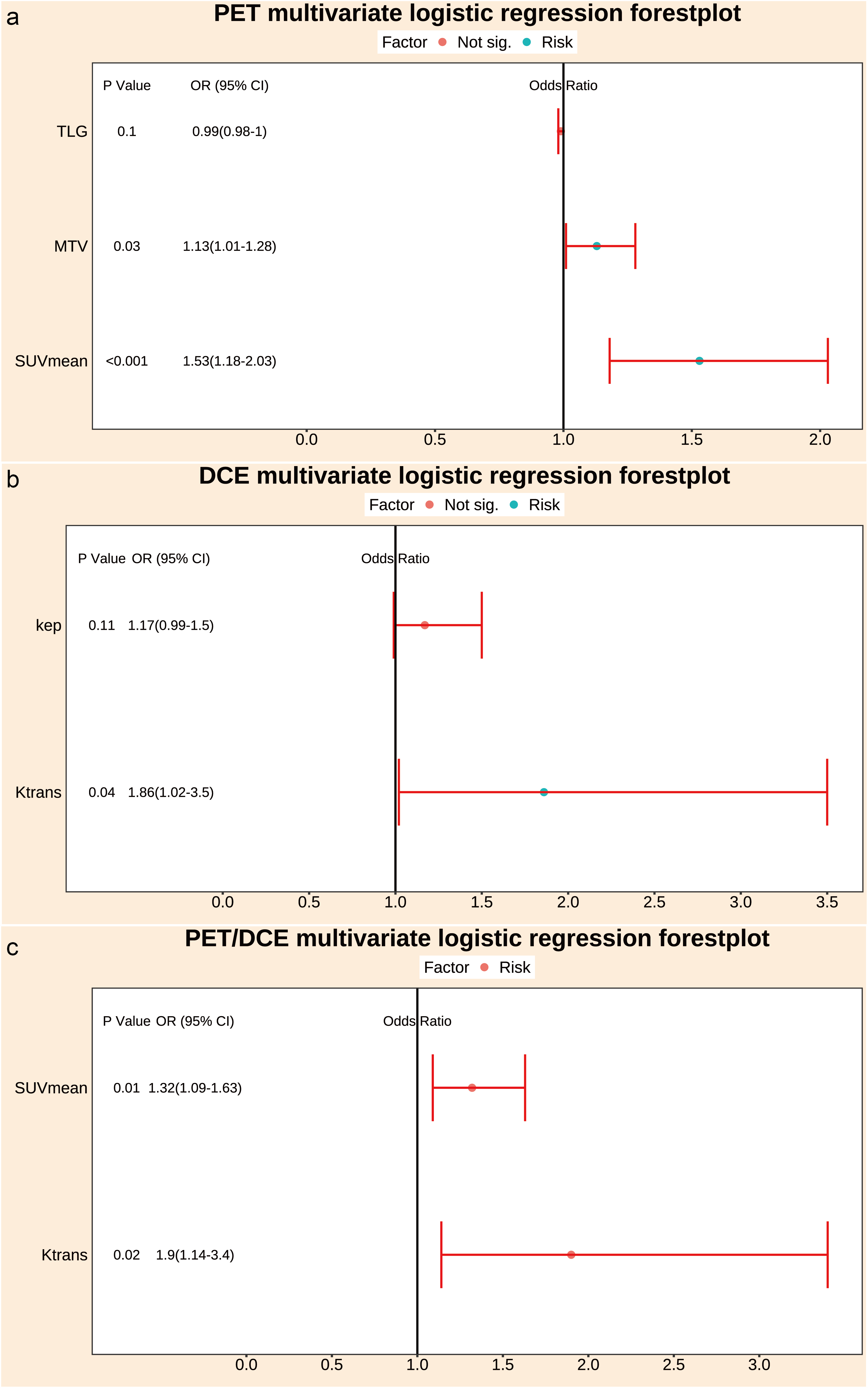


**Supplementary Fig. 2.** Forestplots of PET/DCE-MRI parameters for identifying MMRd. **a** The forestplot of PET parameters contained OR and 95%CI by multivariate logistic regression. **b** The forestplot of DCE-MRI parameters contained OR and 95%CI by multivariate logistic regression. **c** The forestplot of integrating PET/DCE-MRI contained OR and 95%CI by multivariate logistic regression. *MMRd,* mismatch repair deficiency; *MMRp,* mismatch repair proficiency; *OR,* odds ratio; *CI,* confidence interval; *SUV_mean,_* mean standardized uptake value; *MTV,* metabolic volume; *TLG,* total lesion glycolysis; *K_trans,_* transfer constant; *K_ep,_* efflux rate.

**Supplementary Table S2** NRI analysis for PET/DCE-MRI

| Comparisons | PET/DCE vs. PET | | |  | PET/DCE vs.DCE | | |
| --- | --- | --- | --- | --- | --- | --- | --- |
|  | Estimate | Standard Error | 95% CI |  | Estimate | Standard Error | 95% CI |
| NRI for all | 0.04 | 0.10 | -0.14-0.23 |  | 0.13 | 0.12 | -0.11-0.36 |
| NRI for MMRd | 0.00 | 0.08 | -0.16-0.17 |  | 0.15 | 0.11 | -0.08-0.36 |
| NRI for MMRp | 0.04 | 0.05 | -0.05-0.13 |  | -0.02 | 0.04 | -0.11-0.07 |

*NRI,* net reclassification index; *MMRd,* mismatch repair deficiency; *MMRp,* mismatch repair proficiency; *CI,* confidence interval.
